# Influence of a growth hormone transgene on the genetic architecture of growth‐related traits: A comparative analysis between transgenic and wild‐type coho salmon

**DOI:** 10.1111/eva.12692

**Published:** 2018-10-16

**Authors:** Miyako Kodama, Kerry A. Naish, Robert H. Devlin

**Affiliations:** ^1^ Fisheries and Oceans Canada West Vancouver British Columbia Canada; ^2^ School of Aquatic and Fishery Sciences University of Washington Seattle, Washington; ^3^ Present address: Natural History Museum of Denmark University of Copenhagen Copenhagen Denmark; ^4^ Present address: Genome Research and Molecular Biomedicine Department of Biology University of Copenhagen Copenhagen Denmark

**Keywords:** growth, growth hormone, quantitative trait loci, RAD sequencing, risk assessment, salmonids, transgenic

## Abstract

Genetic engineering has been increasingly applied to many commercially important plant and animal species, generating phenotypic changes that are not observed in natural populations and creating genetic interactions that have not experienced natural selection. The degree to and way in which such human‐induced genetic variation interacts with the rest of the genome is currently largely unknown. Integrating such information into ecological and risk assessment frameworks is crucial to understand the potential effects of genetically modified organisms in natural environments. Here, we performed QTL mapping to investigate the genetic architecture of growth‐related traits in nontransgenic (NT) and growth hormone transgenic (T) coho salmon with large changes in growth and related physiology, with the aim of identifying how an inserted transgene might influence the opportunity for selection. These fish shared the same parental genetic background, thus allowing us to determine whether the same or different loci influence these traits within the two groups. The use of over 1,700 loci, derived from restriction site‐associated DNA sequencing, revealed that different genomic regions were linked with growth over time between the two groups. Additionally, the effect sizes of detected QTL appear to have been influenced by the transgene. Direct comparison of QTL between the T and NT fish during two size‐matched periods identified little overlap in their location. Taken together, the results showed that the transgene altered the genetic basis of growth‐related traits in this species. The study has important implications for effective conservation and management of wild populations experiencing introduction of transgenes. Evolutionary changes and their ecological consequences may occur at different rates and in different directions in NT versus T individuals in response to selection. Thus, assessments of phenotypic change, and hence ecological risk, should be determined periodically to evaluate whether initial estimates made with founder strains remain valid.

## 1 INTRODUCTION

Recently, genetic variation has been created in many plant and animal species by humans through genetic engineering (Gaj, Gersbach, & Barbas, 2013; Hsu, Lander, & Zhang, 2014), generating genetic interactions and phenotypic changes not observed in natural populations. Many morphological, physiological, behavioral, and life‐history phenotypes are quantitative in nature and vary continuously in wild populations (Kodama, Hard, & Naish, 2012; Mackay, 2010). The genetic architectures underlying these traits are often complex, and different allelic states of genes, epistasis, and genotype‐by‐sex or genotype‐by‐environment (G × E) interactions are known to influence phenotypic variation (Falconer & Mackay, 1996; Lynch & Walsh, 1998). These factors may do so temporally or continually over the course of development (e.g., Kodama, Hard, & Naish, 2018). As such, the degree to which such anthropogenically introduced variation interacts with the rest of the genome is largely unknown. With a desire to understand potential ecological effects of genetically modified organisms in natural environments, integrating such information into ecological and risk assessment frameworks is crucial to achieve effective conservation and management of wild populations (Devlin, Sundström, & Leggatt, 2015).

Genetic modification of fishes has been underway for more than 30 years, with the primary goals of altering phenotypes for enhancement of production efficiency in aquaculture, applications in basic science, development of strains for the aquarium trade, and control of invasive species (Devlin et al., 2015). Significant potential exists for this technology. However in some cases, such as growth elevation by growth hormone (GH) transgenes, very dramatic phenotypic transformations have arisen. These changes have resulted in public and scientific concern on the potential ecological and evolutionary effects were such animals to enter natural ecosystems. As such, risk assessments are performed that attempt to evaluate how a transgenic organism would interact with ecosystem components to cause undesirable consequences to other species in the ecosystem (Kapuscinski, Hayes, Li, & Dana, 2007). In order to ensure a high degree of reliability, it is critical to know the phenotype and genotype of the transformed organism in detail for risk assessments. For example, effects of transgenes on fitness (survival and reproduction) could affect population size, and behavioral changes may modify the ability of transgenic organisms to utilize or provide resources to other species (e.g., as prey, or as competitive predators).

Assessments of phenotype are usually performed under a single set of laboratory conditions, but it is clear that understanding the stability of that phenotype over time and space is critical to allow prediction of potential effects in the longer term. Data from a transgenic fish species (coho salmon, *Oncorhynchus kisutch*) has shown that traits of genetically modified fish can be highly dependent on environmental conditions (e.g., tank vs. naturalized conditions) and that the phenotypic similarities between transgenic and nontransgenic individuals can vary dramatically among conditions (genotype × environmental responses) (Sundström, Lõhmus, Tymchuk, & Devlin, 2007). In addition to environmental effects, genetic background also appears to affect how a transgene can influence the phenotype. For example, the introduction of a growth hormone transgene into wild strains of rainbow trout (*Oncorhynchus mykiss*) resulted in dramatic elevations of growth rate, whereas introduction of the same transgene into faster‐growing domesticated strains had a much lower effect (Devlin, Biagi, & Smailus, 2001; Devlin, Biagi, Yesaki, Smailus, & Byatt, 2001; Devlin, Sakhrani, White, & Overturf, 2013). In contrast, introduction of the transgene into a partially domesticated strain results in synergistic effects on growth (Devlin, Biagi, & Smailus, 2001; Devlin, Biagi, Yesaki, Smailus, & Byatt, 2001). Similar genetic background effects have been noted in GH transgenic mice (Eisen, Fortman, Chen, & Kopchick, 1993). GH transgenic and domesticated strains possessed similar gene expression profiles, but both were very different from the wild type, revealing how different genes in the genome respond distinctly depending on genetic background (Devlin, Sakhrani, Tymchuk, Rise, & Goh, 2009; Devlin et al., 2013). Modeling studies have revealed how even small effects of background genotype on transgenic organism phenotype can cause very different potential ecosystem consequences (Ahrens & Devlin, 2011).

Experiments examining effects of growth‐related transgenes in different strains of rainbow trout and mice (Devlin, Biagi, & Smailus, 2001; Devlin, Biagi, Yesaki, Smailus, & Byatt, 2001; Devlin et al., 2013; Eisen et al., 1993) suggest that the role of genome‐level influences on phenotypes of transgenic organisms may be common. It is important to extend this knowledge by elucidating the influence that an inserted transgene may have on individual loci underlying complex phenotypes such as growth and to determine whether the same or different loci are involved in these traits in transgenic and nontransgenic organisms. This knowledge will contribute to an understanding of how an inserted transgene might influence phenotypic variability and the opportunity for selection should transgenic individuals enter the wild (Ahrens & Devlin, 2011). Such studies are particularly important in salmonid fishes, where transgenic fish and their wild conspecifics have the potential to interact in common rearing environments (Devlin et al., 2015; Glover et al., 2017).

The effect of transgene insertion on background genetic variation has been examined within few salmonids—limited primarily to coho salmon (*Oncorhynchus kisutch*) and rainbow trout (Devlin et al., 2015). However, research on these species has significant potential to inform risk assessments across the group as a whole, since there is considerable synteny across genomes (Kodama, Brieuc, Devlin, Hard, & Naish, 2014; and references therein) and life histories are frequently shared (Hendry & Stearns, 2004). There are precedents in plants and rodents that genetic background and genetic modifiers can influence phenotypes caused by transgenes (Eisen et al., 1993; Gordon et al., 2008; Saito & Suzuki, 2014; Sher et al., 2014; Suzuki et al., 2008; Zhu, Walker, Boerma, All, & Parrott, 2008). In other cases, QTL have been identified that cause hypermethylation of transgenes with potential effects on expression (Engler et al., 1991; Martin & McGowan, 1995; Sun et al., 2000; Valenza‐schaerly et al., 2001). However, we are not aware of any studies examining transgene–genome interactions at a QTL‐level of resolution, with the specific objective of assessing whether such influences differentially affect wild‐type and transgenic individuals and are of sufficient magnitude to be of potential ecological concern (Ahrens & Devlin, 2011). Very different genetic outcomes are anticipated to arise from selection acting on transgenic and wild‐type organisms in populations, if the influence of genetic modifiers and their mode of action on transgene‐derived phenotypes are sufficiently large. Such outcomes have potential ecological consequences that are difficult to predict at this time (Devlin et al., 2015).

Here, we take advantage of a unique experimental population in coho salmon to investigate the genetic architecture of growth‐related traits in nontransgenic (NT) and growth hormone (GH) transgenic fish (T) using QTL mapping. Individual fish in the population shared the same parental genetic background and experienced large variation in growth and related physiology (Devlin, Biagi, & Yesaki, 2004). A backcross family with a heterozygous transgenic male and wild‐type female was used in the study; therefore, only a half the progeny carried the transgene. We were thus able to examine genomic interactions between transgene and genetic background in NT and T individuals. Specifically, we examined whether QTL affecting variation in growth (body size) of T salmon are shared with, or are distinct from, variation affecting growth in NT salmon.

## 2 MATERIALS AND METHODS

### 2.1 Experimental crosses

Growth hormone transgenic coho salmon were obtained from a strain (M77) containing the OnMTGH1 gene construct stably integrated at a single insertion site in a wild‐type genetic background (Chehalis River, British Columbia, Canada) (Devlin et al., 2004; Phillips & Devlin, 2010). This strain has been extensively studied (Devlin et al., 2015) and results in a large acceleration of growth under aquarium conditions relative to wild type. The strain has been propagated for ten generations via repeated backcrosses of hemizygous transgenic males to wild‐type females obtained from the progenitor strain at each generation to maintain the transgene in a wild genetic background.

**Figure 1:**
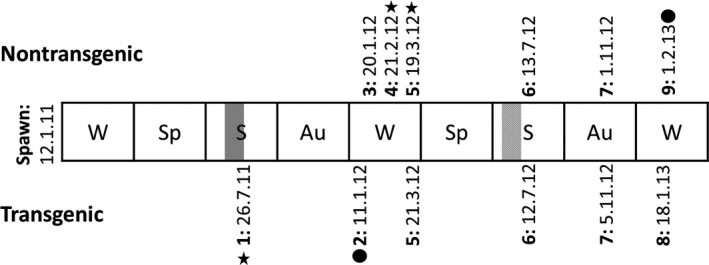
Measurement dates for nontransgenic and transgenic coho salmon. Length and weight were measured at each time period denoted (numbers 1–9, in bold). Periods during which nontransgenic (NT) and transgenic (T) individuals were size‐matched are denoted by a star and a circle; T fish at Time Point 1 were size‐matched to NT siblings at Time Point 4 and 5 for length and weight, respectively (star). T fish at Time Point 2 were size‐matched to NT siblings at Time Point 9 for length (circle). Approximate smolting period for transgenic individuals is denoted by a vertical dark gray bar, for nontransgenic individuals by a hatched light gray bar

A single‐pair backcross family was utilized from a cross performed on January 12, 2011, at the Fisheries and Oceans Canada laboratory in West Vancouver, BC, Canada (Figure 1). The cross was performed between a tenth generation hemizygous male and a wild‐type female. Following hatchery incubation, offspring were reared at densities less than 5 kg/m^3^ and fed with stage‐appropriate commercial salmon feed (Skretting Canada) to satiation seven times per day as fry and three times per day subsequently, ensuring that all fish had access to excess feed. Coho salmon are anadromous; fish were reared in aerated freshwater (±10.5°C) in 170‐L through 3,000‐L tanks prior to smolt (the developmental stage where physiological tolerance to sea water is acquired).

### 2.2 Phenotypic measurements and tissue collection

The wild female × hemizygous transgenic male family used for genotyping contained a 1:1 ratio of nontransgenic (NT) and transgenic (T) siblings. Due to the rapid growth rate of the latter, a bimodal distribution of fish size soon emerged within the family. On July 26, 2011, the fish were separated into small (slow‐growing) and large (fast‐growing) modal groups, and each fish was weighed and measured for length. At this time, the fast‐growing salmon group had achieved smolt status, confirmed based on loss of parr marks and acquisition of a silver body color. These fast‐growing fish were PIT tagged, adipose fin clipped, genotyped to confirm the presence of the transgene (Fitzpatrick et al., 2011), and then transferred for grow‐out to an 18,000‐L tank supplied with oxygenated seawater with temperature ranging from 8 to 12°C. The slow‐growing group contained NT siblings and was monitored for body size by regular assessment of a subsample of the population. Thus, on January 20, 2012, the slow‐growing modal group was PIT tagged and genotyped to confirm the absence of the transgene. At this time, left ventral fins were removed from both NT and the T fish for high‐quality DNA isolation for genotyping.

The objective was to match NT fish to the same body size as when T salmon were first assessed for weight and length in the same freshwater environment on July 26, 2011 (Figure 1). Given the extremely large difference in growth rate between T and NT salmon, age matching would cause fish to be assessed at very large differences in body size. The NT (slow‐growing modal) group was weighed and length measured (at Time Point 4 on Figure 1; indicated with a star) on February 21, 2012, when their median length (11.2 cm) matched the median length of their T siblings (11.4 cm) taken on July 26, 2011 (Time Point 1). The NT group was again weighed and measured on March 19, 2012 (Time Point 5; indicated with a star) when their median weight (19.6 g) matched the median weight of the T group (19.3 g) on July 26, 2011 (at Time Point 1 on Figure 1). Data were collected for the NT group at these two time points since T salmon have a greater condition factor (CF) than NT (i.e., T salmon were heavier for their length than NT salmon). The NT group was transferred to an 18,000‐L seawater tank (separate from the T group) approximately a year later on July 28, 2012, when they had achieved smolt status. During the grow‐out phase of these two groups, weight and length were periodically taken to allow comparison of body‐size phenotypes at later stages (Figure 1). Finally, the length of the NT group was last measured on February 1, 2013, and its median (24.3 cm) most closely matched the median length of their T siblings (31.0 cm) taken on January 11, 2012 (Figure 1; indicated by a circle).

For genotyping and phenotyping, fish were selected (in rank order) from both upper and lower tails of the length distribution of both the NT and T modal groups to enhance the phenotypic divergence among samples analyzed within the resources available. While overall the rank order of fish length and weight were highly correlated for individuals selected for RAD sequencing (nontransgenic *R* ^2^ = 0.75; transgenic *R* ^2^ = 0.88), some exceptions to rank orders of fish existed between length and weight measures, as expected. For the NT group, the small size tail included fish ranging in increasing size rank order from the smallest (7.7 cm; 5.4 g) to the 75th next largest fish (9.9 cm; 10.2 g), and in the large size tail fish ranged in diminishing size rank order from the largest (15.5 cm; 12.8 g) to the 75th next smallest fish (11.5 cm; 15.0 g). For the T group, the small size tail included fish ranging in increasing size rank order from the smallest (8.5 cm; 8.5 g) to the 75th next largest fish (10.9 cm; 16 g), and the large fish size tail included fish ranging in diminishing size rank order from the largest (13.4 cm; 30.3 g) to the 75^th^ next smallest fish (11.8 cm; 22.5 g).

Daily growth coefficients (DGC) at each time interval were calculated for all individuals in each family as follows (Dupont‐Nivet et al., 2010):

DGC = 100 × (final individual weight^1/3^ − initial individual weight^1/3^)/days.

Daily growth coefficients are known to be relatively independent of initial body weight compared to other growth measurements, such as weight gain and specific growth rate (Dupont‐Nivet et al., 2010).

### 2.3 DNA extraction and identification of genetic sex

Genomic DNA was extracted using the DNeasy extraction kit (QIAGEN, Valencia, CA, USA) following the manufacturer's procedures.

Individuals were genotyped using restriction site‐associated DNA (RAD) sequencing (Baird et al., 2008). DNA digestion was performed using the restriction enzyme *Sbf*I, and a 6‐nucleotide barcode was added to each sample for individual identification following protocols described in Baird et al. (2008). A total of 243 individuals were genotyped, comprising 121 and 123 offspring representing the NT and T offspring group, respectively; 36 individuals were pooled into single libraries, and each library was sequenced with 100‐bp single‐read lengths using the Illumina HiSeq 2000 sequencer.

Genetic sex was determined in both NT and T offspring using PCR amplification of a Y‐linked growth hormone pseudogene (Devlin, Biagi, & Smailus, 2001; Devlin, Biagi, Yesaki, Smailus, & Byatt, 2001).

### 2.4 Reference map for QTL detection

The reference map of Kodama et al. (2014) was used to perform sequence calls and detect QTL. Briefly, this map was constructed using two haploid and three diploid families of coho salmon, including the diploid family from the current study; therefore, all the polymorphic markers found in both the male and female meiosis of this family are in the reference map. Both haploid and diploid families were elected in Kodama et al. (2014) because the two types of families provided different levels of information. Specifically, haploid families were used first because they helped (a) discriminate duplicated and nonduplicated loci and (b) allowed correct grouping of markers into linkage groups. After duplicated and nonduplicated markers were identified and markers were correctly grouped and ordered, diploid families were used to add additional markers. Multiple diploid families were elected at this step because a single diploid family can result in erroneous ordering of grouped markers.

Most chromosome arms are syntenic between Salmoninae species, and the combined efforts of genome mapping and karyotyping have permitted alignment of chromosome arms among several species within this subfamily (e.g., Brieuc, Waters, Seeb, & Naish, 2014; Lien et al., 2011; Naish et al., 2013; Sutherland et al., 2016). In the context of linkage mapping, highly conserved synteny across Salmoninae species can be exploited to ensure markers are accurately ordered on the map. To ensure the correct marker orders, Kodama et al. (2014) have adopted this strategy by comparing the marker orders of coho and Chinook salmon reference maps; these species are closely related, sister species with all of the chromosome arms conserved (Phillips & Ráb, 2001). Specifically, Kodama et al. (2014) compared the order of mapped loci in common between these species and showed that the orders for all chromosomes/chromosome arms were highly conserved between the two species, further validating the marker order on the reference map built by Kodama et al. (2014).

Reduced recombination and occasional tetrasomic inheritance are widespread in salmonid males (Allendorf & Danzmann, 1997; Kodama et al., 2014). Because of this, ordering markers in salmonid male is extremely difficult, as a majority of the markers often map to one position on a linkage group (e.g., Kodama et al., 2014). Kodama et al. (2014) addressed this problem by 1) finding polymorphic loci in common between the male parents and the reference map, and 2) ordering grouped markers in the male map based on the known order on the reference map. This step, taken with additional “checks” described above, suggests that the order of the reference map used in the current study was appropriate for QTL detection.

### 2.5 Alignment and genotyping

The sequences were sorted, and the barcodes were used to identify sequences from each individual using the *process_radtags* function implemented in STACKS v.0.9995 (Catchen, Amores, Hohenlohe, Cresko, & Postlethwait, 2011). Because the quality score of sequences decreased beyond 74 nucleotides, sequences were trimmed to 74 nucleotides to eliminate low‐quality sequences. A locus was defined as a 74‐nucleotide RAD sequence for the rest of this study.

Sequences from all individuals were aligned to the reference map of RAD loci for coho salmon developed by Kodama et al. (2014) using BOWTIE v.0.12.9. A maximum of three nucleotide mismatches per locus was allowed to perform alignment. Polymorphic loci with two alleles were identified in each group using STACKS v.0.9995. Genotypes at these loci were determined when alleles were sequenced with a depth greater than 10× per individual. Due to the potential bias toward an excess of homozygous genotypes, genotypes were corrected after running STACKS v.0.9995 with the Python script developed by Brieuc et al. (2014). Specifically, individuals were determined as heterozygotes at a locus if both alleles had a depth of more than two and the total read depth was 10× or greater. Once the genotypes were obtained, polymorphic loci within the NT and T groups were detected, and loci with >50% of progeny genotyped in each group were retained. These loci were further confirmed using the genotypes of the female and male parent.

### 2.6 Statistical description of phenotypes

Two‐sample *F* tests were performed to determine whether phenotypic variances were equal or unequal between female and male offspring for all traits in each group (lengths, weights, and daily growth coefficient). To determine whether the phenotypic means for each trait differed significantly between females and males, independent two‐sample *t* tests were performed with or without equality of variances. Phenotypic correlations between each trait pair in male and female offspring for each group were estimated using Pearson's correlations. All statistical tests were performed in R version 3.0.2 (R Development Core Team 2013).

### 2.7 Transgene mapping

In order to examine whether any QTL were located on a linkage group containing the transgene (details below), markers were grouped via linkage mapping to identify which chromosome harbored the transgene. Specifically, polymorphic loci in the hemizygous father were utilized to perform grouping of the markers. The transgenic locus was coded as heterozygous in the T offspring, and homozygous in the NT offspring, and included in the analyses. Linkage analyses were performed using software for genetic mapping, ONEMAP 2.0–3 (Margarido, Souza, & Garcia, 2007), implemented in R version 3.0.2 (R Development Core Team 2013). Loci were grouped using a log of odds ratio (LOD) score of 3.0 to 7.0 and a maximum recombination fraction of 0.25, and a chromosome containing the transgene was identified.

### 2.8 QTL analyses

QTL mapping was used to compare the genetic architecture between comparable phenotypes in T and NT individuals. To achieve this goal, we compared QTL positions in T and NT groups and examined the relationship between effect size and phenotype at each QTL. Subsequently, QTL expression between the size‐matched periods (Figure 1) was compared.

Quantitative trait locus (QTL) analyses for all traits were performed using single‐, two‐, and multiple‐dimensional QTL models in the R‐based software package, R/qtl (Broman & Sen, 2009; Broman, Wu, Sen, & Churchill, 2003). The reference map for coho salmon developed by Kodama et al. (2014) was used as a framework for the analyses; since this published map was constructed using the individuals in the current study, polymorphic loci observed in this study were captured in the map.

We used the multiple imputation approach to perform quantitative trait loci analyses for all traits (Broman & Sen, 2009), as described in Kodama et al. (2018). In order to accommodate for sex‐specific differences in recombination rates and patterns in salmon, we separately analyzed the loci that were polymorphic in only the father or mother. Missing genotypes given observed marker data were simulated with a step interval of 1 cM, with 516 draws per genotype and assuming a genotyping error rate of 0.01. Genotypic and phenotypic associations were then examined through a series of computational steps. First, a genomewide significance threshold (α = 0.05) LOD score for each trait was determined by 1,000 permutations of genotypic and phenotypic data (Broman & Sen, 2009). Subsequently, single‐QTL analyses were conducted with the *scanone* function implemented in R/qtl. To explore the effect of offspring sex on QTL expression, offspring sex was included as an additive or interactive covariate in the analyses for all traits (Broman & Sen, 2009). Further testing was performed with QTL‐linked markers as additive covariates in a single‐QTL scan to detect additional loci with modest effects; here, QTL that exceeded the 5% genomewide significance thresholds in the initial computation were used as an additive covariate in the model. This process was repeated until no new QTL were identified. Once all QTL were identified in a single‐QTL scan, interactions between QTL were tested by performing a two‐QTL genome scan to investigate the presence of epistatic interactions with the *scantwo* function. Finally, significant QTL and QTL × QTL interactions detected by the single‐ and two‐QTL analyses were fitted into a multiple QTL model with offspring sex as a covariate. Improved estimates of the QTL locations were obtained with the *refineqtl* function implemented in R/qtl. The improved estimates of the QTL locations were used to fit a multiple model containing all QTL effects, as well as significant QTL × sex and QTL × QTL interactions. Any insignificant QTL or interaction terms at α = 0.05 genomewide thresholds were removed, and the location of remaining QTL was refined in an iterative approach until only significant terms remained in the model. For all QTL significant at *p* ≤ 0.05, the 95% Bayes credible interval (CI) was obtained using the *bayesint* function. Percentage of phenotypic variance explained (PVE) by each QTL was estimated using the *fitqtl* function, and QTL of major or minor effect (PVE of over or less than 15%, respectively) were identified.

### 2.9 Gene annotation

QTL‐associated loci explaining large effect sizes, along with their flanking markers on the genome map, were aligned to the reference genome for coho salmon (Accession PRJNA378663, Okis_V1; https://www.ncbi.nlm.nih.gov/assembly/GCF_002021735.1), which in turn has been annotated using data from Kim, Leong, Koop, and Devlin (2016). Alignments were performed using Bowtie 2 (v.2.2.9) (Langmead & Salzberg, 2012) with default parameters. The confidence limits for QTL‐linked markers were large (Results section); therefore, the names and products of genes falling within 1 Mb of QTL‐linked markers were reported.

Potential candidate genes for the QTL were identified using mRNA sequences from two studies that have reported differential gene expression between transgenic and nontransgenic coho salmon (Devlin et al., 2009; Kim, Leggatt, Chan, & Devlin, 2015). These sequences were aligned to the transcriptome data of Kim et al. (2016) using a BLASTn search (Altschul, Gish, Miller, Myers, & Lipman, 1990) with default parameter settings and an e‐value threshold of 1 × 10^−10^. Protein sequence identifiers (db_xref) were then cross‐referenced to determine whether differentially expressed genes from the earlier studies fell within the annotated regions.

## 3 RESULTS

### 3.1 Phenotypic trait measures and statistical description of traits

Both NT and T groups, comprising siblings from the same family that differed in the presence or absence of the transgene, were measured for phenotypic traits associated with growth at six time points, spanning from July 26, 2011, to February 1, 2013 (Figure 1).

The NT group was measured for 17 phenotypic traits associated with growth: fork length and body weight at six time points from January 20, 2012, to February 1, 2013, and daily growth coefficients across five time intervals (Figure 1). The T group was also measured for 17 phenotypic traits associated with growth: fork length and body weight at six time points from July 26, 2011, to January 18, 2013, and daily growth coefficients across five time intervals (Figure 1).

We observed sex‐specific differences in phenotypes in both NT and T groups. Means and variances between female and male offspring in the NT group differed for lengths and weights measured at three to four time points (from January 20, 2012, to July 13, 2012; Table 1) out of six time points. Means and variances for the majority of the traits differed significantly between female and male offspring in the T group (Table 1).

**Table 1:** Summary of the number of male and female offspring, as well as the phenotypes (length, cm; weight, g; daily growth coefficient, DGC) measured in male and female offspring from each transgenotype group

Group	Number of offspring		Phenotype	Male Offspring		Female offspring		*F* test *p*‐value	*T* test *p*‐value (equal or nonequal variance)
	Male	Female		Mean	*SD*	Mean	*SD*		
NonTransgenic	50	71	Length_TP_3	10.98	1.32	10.80	1.26	0.70	0.44
			Length_TP_4	11.49	1.26	11.26	1.72	0.03	0.40
			Length_TP_5	12.41	1.22	11.86	0.89	0.02	0.01
			Length_TP_6	15.83	2.21	14.70	1.23	0.00	0.01
			Length_TP_7	20.72	2.21	20.16	2.53	0.51	0.38
			Length_TP_9	23.68	3.09	24.03	2.75	0.57	0.70
			Weight_TP_3	14.71	5.36	13.52	4.09	0.04	0.19
			Weight_TP_4	17.84	6.34	15.70	4.40	0.01	0.05
			Weight_TP_5	22.16	7.14	19.31	4.55	0.00	0.02
			Weight_TP_6	43.55	23.63	32.81	9.64	0.00	0.01
			Weight_TP_7	118.04	37.02	104.77	36.75	0.95	0.18
			Weight_TP_9	182.14	64.35	183.53	60.76	0.75	0.94
			DGC_TP_3_4	0.45	0.29	0.38	0.34	0.31	0.27
			DGC_TP_4_5	0.75	0.35	0.67	0.42	0.19	0.27
			DGC_TP_5_6	0.54	0.28	0.44	0.19	0.01	0.06
			DGC_TP_6_7	1.24	0.34	1.33	0.34	0.96	0.30
			DGC_TP_7_9	0.91	0.33	0.90	0.26	0.29	0.93
Transgenic	64	58	Length_TP_1	10.67	1.03	11.81	0.79	0.04	0.00
			Length_TP_2	29.42	2.72	32.34	1.69	0.00	0.00
			Length_TP_5	30.22	2.75	32.17	4.89	0.00	0.01
			Length_TP_6	37.69	4.47	40.67	4.19	0.66	0.00
			Length_TP_7	48.30	4.61	49.77	3.92	0.27	0.09
			Length_TP_8	51.05	4.85	52.04	4.04	0.29	0.35
			Weight_TP_1	16.72	4.58	21.17	3.87	0.20	0.00
			Weight_TP_2	368.94	98.15	485.59	63.48	0.00	0.00
			Weight_TP_5	376.95	102.21	470.54	101.63	0.97	0.00
			Weight_TP_6	780.28	243.92	958.11	204.03	0.23	0.00
			Weight_TP_7	1,710.87	460.67	1,909.47	408.41	0.41	0.03
			Weight_TP_8	1,689.41	497.96	1,992.21	491.05	0.94	0.01
			DGC_TP_1_2	2.71	0.28	3.01	0.15	0.00	0.00
			DGC_TP_2_5	0.02	0.24	−0.26	1.60	0.00	0.22
			DGC_TP_5_6	1.71	0.47	1.79	0.32	0.01	0.32
			DGC_TP_6_7	2.30	0.54	2.19	0.36	0.01	0.24
			DGC_TP_7_8	−0.22	0.60	0.17	0.71	0.29	0.01

Note

Mean and standard deviation (*SD*) for male and female, as well as the *p*‐values obtained from the *F* tests and *t* tests assuming equal or nonequal variance, are shown. Calculations are based on sequenced individuals.

Almost all lengths and weights were significantly and positively correlated with each other in both male and female progeny in each group (Data S1). In contrast, the degree, direction, and significance of correlations for daily growth coefficients were mixed in both groups.

### 3.2 Genetic markers

NT and T groups were derived from the same family, and thus, comparable numbers of loci were observed in each group. There were fewer polymorphic loci in the hemizygous father compared to the wild mother (Data S2). The small difference in the number of polymorphic markers between the NT and T groups was observed due to the threshold of missing data; the NT and T groups had 1,784 and 1,741 biallelic loci, respectively, that were polymorphic in the female parent with known position on the coho reference map (Kodama et al., 2014). Of these markers, more than 95% (1,706 loci) were in common across the two groups and were distributed across all linkage groups (Data S2). The NT and T groups had 903 and 908 biallelic loci, respectively, that were polymorphic in the male parent with known position on the coho reference map; of these, >95% of loci (864 loci) were in common across the two groups. Polymorphic loci in the hemizygous father were distributed across all linkage groups except for the linkage group Co13, which only harbored two polymorphic loci in the transgenic group (Data S2).

### 3.3 Transgene mapping

The transgene mapped to a putative p arm of a metacentric chromosome, Co13. This chromosome arm designation is based on the size of the linkage group in Kodama et al. (2014). Co43847_Co13a was the closest marker linked to the transgene, and this marker and the transgene were 9.45 cM apart.

### 3.4 QTL analyses within T and NT groups

The QTL analyses performed across the two groups separately relied on comparable numbers of individuals—121 offspring representing the NT offspring group and 122 representing the T group. There were only two size‐matched stages (Figure 1; indicated with a star and circle): the first made with fish in freshwater (star) and the second in sea water after smoltification (circle). Therefore, direct comparative descriptions were limited to these periods only, but there are general observations that may be made first.

**Figure 2:**
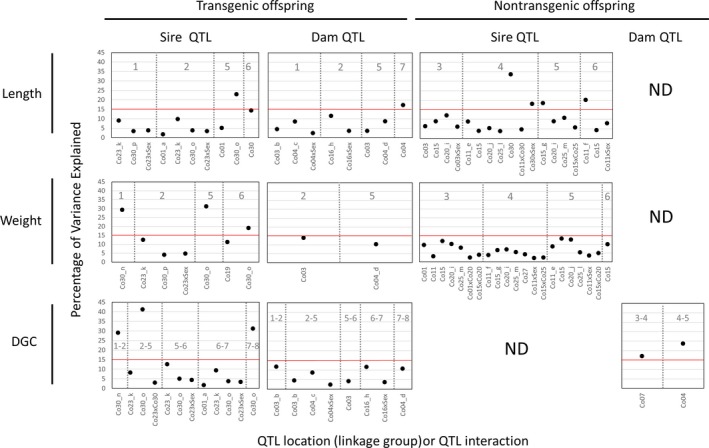
Comparison of QTL positions and percentage of variance explained between transgenic (T) and nontransgenic (NT) siblings, produced from a cross between a wild‐type dam and a hemizygous sire with a growth hormone transgene. Shown are growth‐related QTL mapped across different time periods (in gray, separated by vertical dashed lines), using segregating markers from the sire and the dam. Positions of QTL are denoted by coho salmon linkage groups (Co01‐30, Kodama et al., 2014); QTL sharing the same nearest marker (across traits or different time periods) are designated by “CoXX_letter” (e.g., “Co23_k”). QTL detected with a unique marker is simply denoted as CoXX. “ND” indicates that no QTL were found. Size‐matched periods between T and NT groups are periods 1 and 4 for length, 1 and 5 for weight, and 2 and 9 for length. Full details are provided in Data S3

First, 30 significant QTL for length, weight, and daily growth coefficient were detected in the NT group over the entire experimental period at the α = 0.05 genomewide thresholds (Figure 2; Data S3A). The majority of the QTL were detected using the segregation information from the sire, and only two QTL were found using the information from the dam. This latter result is likely an outcome of the higher recombination rate in female salmonids (Sakamoto et al., 2000). In the T group, 37 significant QTL were detected across the experiment (Figure 2; Data S3B). In contrast to the NT group, comparable numbers of QTL were detected using the segregation information from the dam and sire (14 and 23, respectively). The locations of QTL detected with the dam generally spanned a narrower region of the chromosome (Data S4). No QTL were linked to the linkage group harboring the transgene; for reasons, we discuss below.

Second, a majority of the QTL were of modest effect across both groups, ranging from 3% to 15% (Figure 2; specific values in Data S3A and B). There were 13 QTL of major effect (PVE of over 15%) overall: 5 and 8 QTL in the NT and T groups, respectively. In the NT group, two and three QTL of major effect were detected using the segregation information from the dam and sire, respectively. In the T group, a majority of the major effect QTL (7 out of 8) were detected using the segregation information from the sire, and all 7 QTL were mapped to Co30; of these, five QTL of major effect mapped to one specific region of Co30 (55‐78 cM), sharing the same closest linked marker (“Co30_o” in Figure 2). Two additional major effect QTL mapped to another region of Co30 (55‐166 cM), sharing the same closest linked marker (“Co30_n” in Figure 2).

Third, certain QTL were consistently observed across different time points in the study (Figure 2; shared QTL location designated by “_letter,” e.g., “Co30_o”). Notably, the markers on Co23 and Co30 were repeatedly linked with multiple lengths, weights, and DGCs in the T group, and all of these QTL were detected using the sire. Similarly, the same markers were linked with QTL on Co03 and Co04 across multiple time points, and these QTL were detected with the dam. In the NT group, the same markers on Co11, Co15, Co20, and Co25 were linked with numerous QTL for traits measured across time; these QTL were all detected using the sire.

Fourth, QTL × QTL interactions were observed for five traits in the NT group (Figure 2; Data S3A,B) and one trait in the T group. These results indicate the role of epistatic interactions affecting traits measured; however, in all cases QTL × QTL interactions were of minor effect, each explaining around or less than 5% of PVE.

Finally, Sex × QTL interactions were also detected for several QTLs in both NT and T groups, indicating sex‐dependent genetic effects (Figure 2). The effect sizes of these interactions were comparable across the two groups, explaining less than 10% of PVE, except for length measured at one time point in the NT group (Length_TP_4).

### 3.5 Comparative analysis of QTL location and effect sizes at size‐matched time points

**Figure 3:**
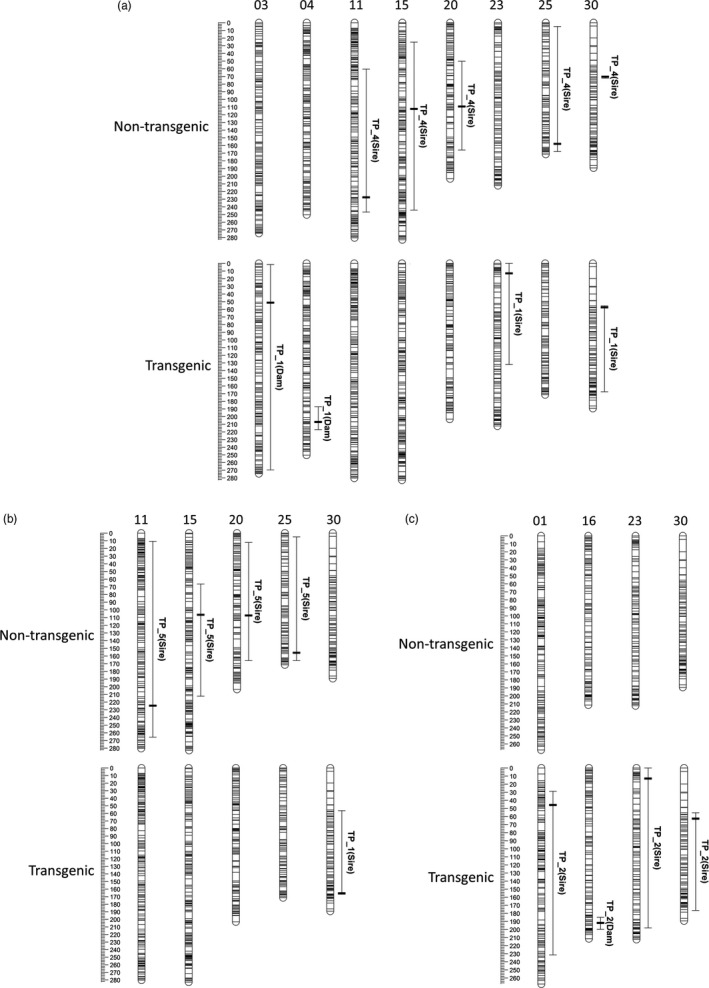
Quantitative trait loci (shown as 95% Bayes credible intervals) for size‐matched traits (TP = Time Point, see Figure 1). QTL underlying the following comparisons in nontransgenic (NT) and transgenic (T) groups, respectively, are shown based on the reference map created by Kodama et al. (2014): (A) Length_TP_4 (NT) and Length_TP_1 (T), (B) Weight_TP_5 (NT) and Weight_TP_1 (T), and (C) Length_TP_9 (NT) and Length_TP_2 (T). QTL detected in the NT and T groups are shown as top and bottom rows, respectively. Only linkage groups with significant QTL (detected in either family) are shown, and linkage groups are named accordingly to the previously published map (Kodama et al., 2014). The marker associated with each QTL is shown by a horizontal bar. The parent (dam or sire) in which the corresponding QTL were detected is shown in parentheses

The NT and T fish were size‐matched at two time points (Figure 1): the first in freshwater (denoted with a star in Figure 1) and the second in seawater after smoltification (denoted with a circle in Figure 1). The groups were length‐matched at Time Point 4 for the NT fish and at Time Point 1 for the T fish. QTL segregating in the dam were detected in the T group only (Figure 3.A); these QTL were not detected in the NT group. QTL segregating in the sire were detected in both groups (five and two in NT and T groups, respectively), but only shared one QTL that mapped to Co30 (Figure 3.A); interestingly, this QTL was of large effect (~30%) in the NT fish, while it was of small effect (~3%) in the T fish (Data S3A,B).

The groups were weight‐matched at Time Point 5 for the NT fish and Time Point 1 for the T fish (Figure 3.B). One QTL of large effect (~30%) was detected on Co30 in the T group, while four QTL of modest effect sizes (5%–13%) were detected in the NT fish; none of these QTL were shared between the two groups (Figure 3.B).

Finally, the two groups were length‐matched again at Time Point 9 for the NT fish and at Time Point 2 for the T fish. No QTL were detected in the NT group (Figure 3.C). In the T group, 3 and 1 QTL were detected using the segregation information from the sire and dam, respectively; all QTL were of modest effect (1%–11%; Data S3B).

### 3.6 Gene annotation

QTL‐associated markers with large effect sizes were identified on linkage groups Co04, Co11, and Co30. We therefore aligned these markers along with other QTL‐associated markers falling on the same linkage groups.

Three sire‐associated QTL markers on Co30 were linked within 1 Mb of each other (Data S5); one marker (Co50943_Co30) was of large effect size and two were expressed repeatedly within the T group (Co50943_Co30 and Co30795_Co30). All markers fell near or within genes (*myc* and *adcy8*; Data S5). The remaining QTL of large effect size Co59610_Co30, also expressed temporally within the T group, was located within a gene (*LOC109874585*, product “ETS‐related transcription factor Elf‐1‐like”). A total of 17 differentially expressed genes from the studies of Devlin et al. (2009) and Kim et al. (2016) aligned to Co30; one (*LOC109874801*) was linked to Co59610_Co30 at an approximate distance of 500 kb. The product of this gene has been inferred as “complement C3‐like.” This gene was expressed in the pituitary in the comparative analysis of Kim et al. (2016) and may play a role in inducing smooth muscle contraction.

Two separate Dam QTL of large effect size on Co04 were detected in the NT (Co17102_Co04a) and T groups (Co09937_Co04b), respectively. The latter was linked to two temporally expressed loci within a 3 MB region (also in the T group; Co64498_Co04b and Co28666_Co04b). All three loci were located within genes. However, only one of the 21 differentially expressed genes aligned with this chromosome was located within the annotated regions, and this locus (*LOC109889390*) was linked to the QTL of large effect size expressed in the NT group (Co17102_Co04a). The locus was expressed in the liver in Devlin et al. (2009) and encodes a “small EDRK‐rich factor 1‐like” protein that is ubiquitous, but the function is unknown.

Finally, three Sire QTL‐linked markers on Co11 were detected in the NT group: Co49885_Co11a, Co58715_Co11b, and Co58273_Co11a. Each was located in separate unlinked regions of the chromosome (Data S5) and was located within genes (in order—*LOC109898876*;* topbp1*;* LOC109899855*). The latter two QTL markers were temporally expressed and the last marker was of large effect size. A total of 31 differentially expressed genes aligned with this chromosome. The gene *LOC109899444* “cytochrome P450 2F3‐like” was located within 55 kb of the temporally expressed locus Co58715_Co11b and may be involved in oxidative degradation of compounds such as environmental toxins. This gene was expressed in the liver in the analysis of Devlin et al. (2009). A second, *LOC109899847* “mitoferrin‐2‐like” was within 100 kb of the QTL locus of large effect, has been designated as a mitochondrial carrier protein, and was differentially expressed in muscle tissue (Devlin et al., 2009).

## 4 DISCUSSION

The aim of this study was to determine whether the insertion of a growth hormone transgene influenced the genetic architecture underlying growth‐related traits in coho salmon relative to nontransgenic individuals. This aim was achieved by mapping QTL in 243 offspring that differed only in the insertion of the transgene, by comparing QTL at shared size‐matched stages and by examining consistency in temporal expression during their growth period. Broadly, QTL were observed using the segregation information from the sire in both the transgenic (T) and nontransgenic (NT) offspring, but no QTL in common between T and NT were found except for one QTL on Co30 (Length_TP_4 and TP_1 in NT and T groups, respectively). QTL based on the segregation information from the dam were detected primarily in the T group but rarely in the NT group. QTL of large effect sizes were observed in both NT and T offspring, but were more frequent in the latter. Five out of eight large effect QTL in the T fish shared the closest linked marker on Co30 and were detected using the sire. Generally, the same markers were linked with QTL for traits measured across the course of development in each group; none of these markers were shared between the groups. Comparison between size‐matched traits at two time points showed that almost none of the QTL were shared between NT and T offspring. Finally, comparison between size‐matched traits at two time points showed that almost none of the QTL were shared between NT and T offspring. We conclude that the insertion of the transgene affects both the expression and the effect sizes of QTL involved in growth‐related traits, thus influencing the phenotypic variance and opportunity for differential selection between T and NT individuals.

The study has several underlying assumptions. First, transgenic fish have a rapid growth rate and mature earlier; therefore, temporally matched measures would likely detect different traits between T and NT to a greater degree than would size‐matched traits explained simply by body‐size effects. We note that length and weight in the T fish in the first comparison overlapped with two separate periods in the NT fish, implying that shape varies between the two groups. This result may reflect influences arising from age as well as the transgene. It is therefore possible that we may have measured different traits. However, differences in physiology (and its genetic control) are precisely what the present study is seeking to assess. For example, T coho salmon undergo smoltification within their first few months of life (postfirst feeding) in freshwater, compared to approximately 12–14 months for wild‐type coho salmon, and have enhanced osmoregulatory capability for a given size compared to wild type (Bystriansky et al., 2017; Devlin et al., 2000). Such physiological differences are very likely to also influence growth and do so differently between T and NT salmon. Second, we assumed that there was sufficient power to detect relevant growth‐related QTL within each experimental group. The lack of detection of QTL in one group does not mean that it is not present in the other. However, it is important to point out that the power of detection was very similar in both groups, since 1) the sample sizes were comparable, and 2) the set of polymorphic loci used to detect QTL as the NT and T fish were derived from one family and thus comparable. Therefore, the analyses likely identified key differences between the groups. Third, we recognize that the ability to detect QTL across sexes differed due to the number of polymorphic markers and because female salmonids have higher recombination rates than males (Kodama et al., 2014; Sakamoto et al., 2000). Therefore, linkage between markers and QTL segregating in the females is be expected to be lower than in males and likely explains why there was a lower detection rate in the former. Again, unreported QTL in females compared to males do not mean that such QTL are not present. Fourth, we acknowledge that the present results may not predict the specific QTL that may be expressed in other crosses. The analyses relied on the segregation of alleles in the parents of the cross. Nevertheless, the value of the study relies on the comparative nature of the experimental design; the T and NT offspring shared the same genetic backgrounds on average; and the analyses revealed key differences in QTL expression between the two groups at matched body sizes.

The transgene in the M77 strain was located on a metacentric chromosome, Co13 (Kodama et al., 2014), but no QTL were attributed to this linkage group. The location of the insertion extends the in situ hybridization study of Phillips and Devlin (2010), which identified a medium‐sized metacentric chromosome as the site of the transgene. In the present study, the precise position of the transgene could not be identified, due to the low number of polymorphic loci observed on this linkage group (Data S2). Comparable numbers of polymorphic markers were observed for other linkage groups identified in the coho genome (Kodama et al., 2014) in NT and T fish; therefore, the reduced polymorphism may be explained by the breeding history of the M77 strain. A series of backcrossing events with only one or a few transgenic parents contributing to subsequent generations has likely resulted in a selective sweep for the transgene‐containing region in Co13. The low number of polymorphic markers in T fish might explain diminished power to detect QTL (Data S3B), and why it was not possible to detect QTL interactions of Co13 with the transgene.

Since all fish in this study were derived from shared genetic backgrounds, a lack of common QTL detected between T and NT groups likely arose from interactions between the transgene and other loci in the genome affecting growth. Therefore, the QTL involved in physiological pathways shaping growth may differ between NT and T fish (see osmoregulation example mentioned above). Alternatively, if the QTL are shared (and were not detected in one group due to the power of the study), the transgene likely influences the effect sizes of these loci through interactions we were unable to study. These findings support previous observations of large differences in expression levels for genes involved in physiological and endocrine pathways between NT and T fish of the same strain, based on microarrays and RNA‐seq (Devlin et al., 2009; Rise et al., 2006). In all studies, a wide range of differences in growth, body shape, and feeding behavior were observed between NT and T fish in captivity (Devlin et al., 2015), suggesting that growth differences arose from the physiological, morphological, and behavioral differences between NT and T fish.

One of the most interesting results of this study was that thirteen QTL were localized on Co30 in T fish, whereas only one QTL was found on this linkage group in NT fish. More than half of the QTL located on Co30 in the T group were of larger effect sizes (>15% of PVE). Current evidence based on genome mapping suggests that Co30 is the sex chromosome in this species (Kodama et al., 2014). However, these QTL did not overlap with the sex locus, which maps to the centromere (0 cM). On the other hand, we have previously reported a number of QTL on Co30 in three crosses based on aquaculture strains (Kodama et al., 2018); one for age at sexual maturity, two QTL for length and weight measured at two time points, and one for daily growth coefficient. These six QTL overlapped with those detected in the current study, although QTL from the two studies were linked with different markers. It appears that the transgene may have influenced the effect size of one or more QTL on this chromosome that may be involved in growth‐related traits during the development of coho salmon, generally. Annotation of the QTL‐associated regions on the coho salmon genome revealed close linkage between three of the markers (all within a 2 Mb region) detected in the T offspring. Comparison with earlier data on differential gene expression (Devlin et al., 2009; Kim et al., 2015) revealed one candidate gene associated with a fourth QTL marker that was repeatedly expressed in the T offspring. The mRNA from this gene (complement‐C3) had been isolated from pituitary tissue and may play a role in smooth muscle contraction. We also note the overlap between temporally expressed QTL on Co03 and Co23 in the T fish and previously reported growth‐related QTL (Kodama et al., 2018), again suggesting that the transgene influences the expression of key loci involved in coho salmon development relative to the nontransgenic fish.

The transgene may interact with phenotypic sex, and its insertion may have even changed how phenotypic sex interacts with the genome, although we were unable to detect interactions between the transgene and sex within the T progeny due to the reduced variation around this locus. While sex × QTL interactions were widespread and equally as common between NT and T fish, none were shared. We have previously reported that sex × QTL interactions play an important role on the growth and maturity in this species (Kodama et al., 2018). While epistatic QTL × QTL interactions were relatively common in NT fish (multiple QTL x QTL interactions observed for five growth‐related traits), such an interaction was only observed once in T fish. In NT fish, the effects of QTL × QTL interactions were modest, explaining <5% of phenotypic variance (Data S3A). However, again, we were unable to study the most important interaction—that between the transgene and the other QTL. The transgene is likely to be the dominant influence on growth in T fish; therefore, detection of significant QTL × QTL interactions between other loci may have been restricted in T fish due to their modest relative effect, and such interactions may only be detected if families larger than those employed in the present study were utilized.

This study provides important insights into understanding how a transgene may influence a species’ response to selection in natural environments. The phenotype of GH transgenic salmon can clearly be influenced by background genetic variation, and as such, selection acting on this variation in nature would be expected to alter the phenotype of T fish in directions that would improve their fitness. There is significant evidence from this study that the transgene alters the effect size of key loci in the coho salmon genome. While response to selection is often ascribed primarily to additive genetic variation (Hill, Goddard, & Visscher, 2008), epistatic variation can act to release additive variance by influencing effect sizes QTL (Forneris, Vitezica, Legarra, & Pérez‐Enciso, 2017), thus accelerating the rate of evolution (Paixão & Barton, 2016). Selection may direct phenotypes to faster or slower growth, depending on whether T fish have enhanced or diminished fitness relative to wild type. It seems likely that the T phenotype could shift over time from when the strain was originally synthesized (Ahrens & Devlin, 2011). It is the phenotype of T fish (e.g., aggressive feeding behavior; predation susceptibility) that would drive potential effects on other ecosystem members through processes such as competition or predation. As such, understanding how the phenotype of T fish can change from selection over time is critical to improve risk estimates. If the selection on background genotypes and the alteration of phenotype is substantial, the ecological effects of T fish may change, complicating risk assessments. Further, if different genomic regions affect variation in growth in NT and T fish, or if the effect sizes of specific loci in a pathway are increased, these regions may respond distinctly to various selective pressures. Different genetic variation may consequently arise in populations as a result. Such phenomena could cause genetic conflict if NT and T fish interbreed; for example, if the selected background variation improving the fitness of T fish is not the best background genotype for fitness of NT fish (Ahrens & Devlin, 2011). Identifying the mechanism by which modifiers affect transgenic traits will be important to improve our ability to understand the basis of phenotypic variation in genetically modified organisms with application to ecological risk assessments and for selective breeding strategies using transgenes as sources of variation. We do not know how the loci identified in the present study act to modify the GH transgene's influence on phenotype. However, we also note that the transgene is itself tandemly repeated at a single locus and has inserted into a centromeric region of the chromosome rich in repetitive DNA (Phillips & Devlin, 2010; Uh, Khattra, & Devlin, 2006). As such, its expression may be under the influence of heterochromatin and be experiencing position‐effect variegation or hypermethylation, as seen for transgenes in other systems. Both of these influences on gene expression are known to be affected by genetic modifiers (e.g., Sinclair, Mottus, & Grigliatti, 1983; and see Introduction).

This study also has important implications for aquaculture should GH transgenic strains be used in the future. Because multiple genomic regions other than the transgene have been shown to play an important role in determining growth of T fish, it is important to consider the level of genetic variation in these regions to determine whether selection of desired phenotypes could still occur when performing selective breeding. Indeed, while T fish have been strongly growth enhanced, the strains developed to date have been generated in wild and domesticated genetic backgrounds that may not be optimal for growth physiology during GH overexpression. Genetic selection for elevated growth in aquaculture strains of salmonids has been very successful (Gall & Huang, 1988; Gjedrem, 2000; Hershberger, Myers, Iwamoto, Mcauley, & Saxton, 1990) indicating a substantial level of variation for this trait exists in wild populations. The growth rate of T fish in nondomesticated wild genetic backgrounds may also be subject to further selection from the original phenotype found in the founder strains. The present data would suggest that such selection in T fish would occur at loci that did not play a major role in growth regulation in NT fish. Thus, marker‐assisted breeding strategies for T organisms should ideally utilize markers identified within the T background.

The results of this study are based on data obtained from one family: a single‐pair backcross between a hemizygous male of the M77 GH transgenic strain and a wild‐type female. As NT and T fish were derived from the same family, the genetic background in the two groups was the same; therefore, this experimental setting allowed us to sensitively examine the effect of the transgene and its interaction with the genome on the growth‐related traits. This approach was chosen to improve the sensitivity of detecting transgene‐background genotype interactions compared to a wider population analysis. Nevertheless, further studies including multiple families in different environments would be desired if one aims to assess the generality of our findings, or characterize how a transgene affects a population. That said, this study using NT and T coho salmon offers an interesting model to examine genomic interactions between a transgene and genetic background; the results demonstrated that genetic changes with powerful effects on phenotype (i.e., growth hormone transgene) strongly altered the genetic basis of growth‐related traits in this species. Different genomic regions were involved in determining growth over time, and hence, evolutionary changes and ecological consequences may occur in different directions in NT vs. T fish in response to selection within the complexities of environmental conditions in nature. This study provided important insights into genetic complexities associated with understanding phenotype of T fish and has provided the foundation for future efforts in localizing candidate genes shaping growth regulation in T fish. The data also have important implications for assessments of the stability of phenotypes of T strains which is critical for determinations of potential ecological risks should such animals ever escape into natural environments.

## CONFLICT OF INTEREST

The authors declare that they have no competing interests in relation to this manuscript.

## DATA ARCHIVING

Data available from the Dryad Digital Repository: https://doi.org/10.5061/dryad.fs4b1b3.
