# Supplementary material for: Influence of a growth hormone transgene on the genetic architecture of growth‐related traits: A comparative analysis between transgenic and wild‐type coho salmon
Source: Evol Appl. 2018 Oct 16;11(10):1886–900. doi: 10.1111/eva.12692 (PMC6231474; doi:10.1111/eva.12692)
Supplement: Supplementary file 1 [file EVA-11-1886-s001.pptx]

## Slide 1
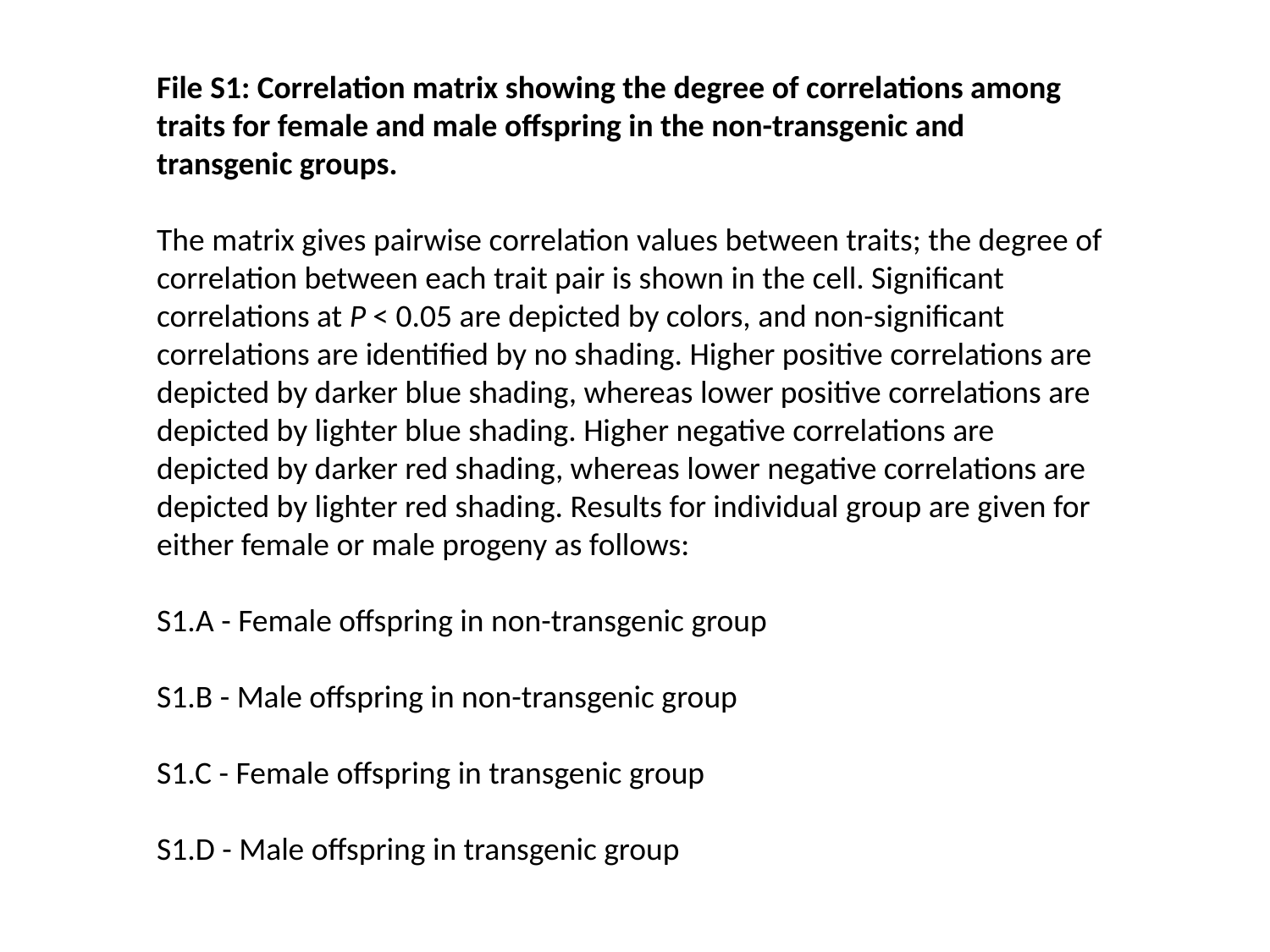

File S1: Correlation matrix showing the degree of correlations among traits for female and male offspring in the non-transgenic and transgenic groups.
The matrix gives pairwise correlation values between traits; the degree of correlation between each trait pair is shown in the cell. Significant correlations at P < 0.05 are depicted by colors, and non-significant correlations are identified by no shading. Higher positive correlations are depicted by darker blue shading, whereas lower positive correlations are depicted by lighter blue shading. Higher negative correlations are depicted by darker red shading, whereas lower negative correlations are depicted by lighter red shading. Results for individual group are given for either female or male progeny as follows:
S1.A - Female offspring in non-transgenic group
S1.B - Male offspring in non-transgenic group
S1.C - Female offspring in transgenic group
S1.D - Male offspring in transgenic group

## Slide 2
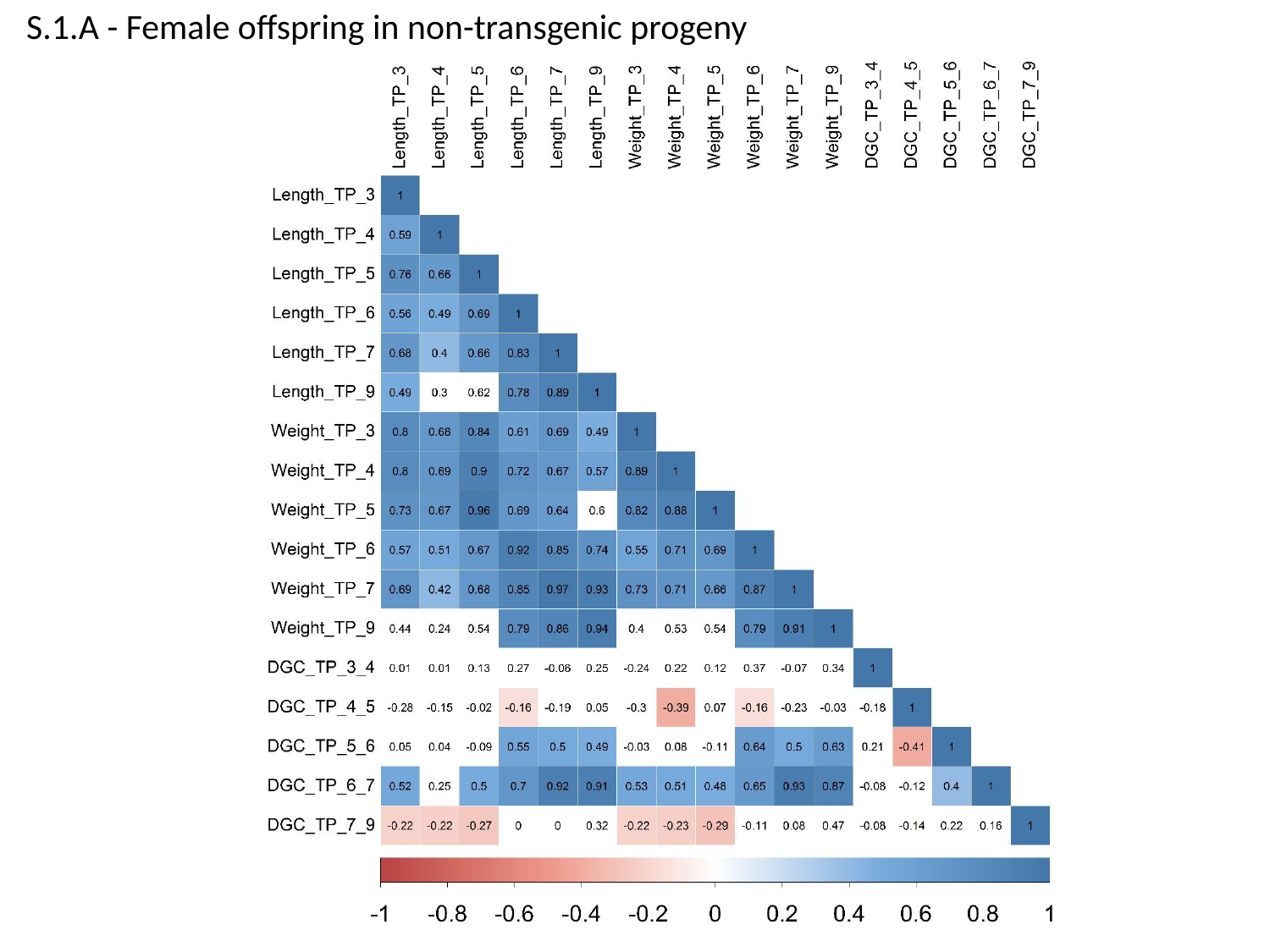

S.1.A - Female offspring in non-transgenic progeny

## Slide 3
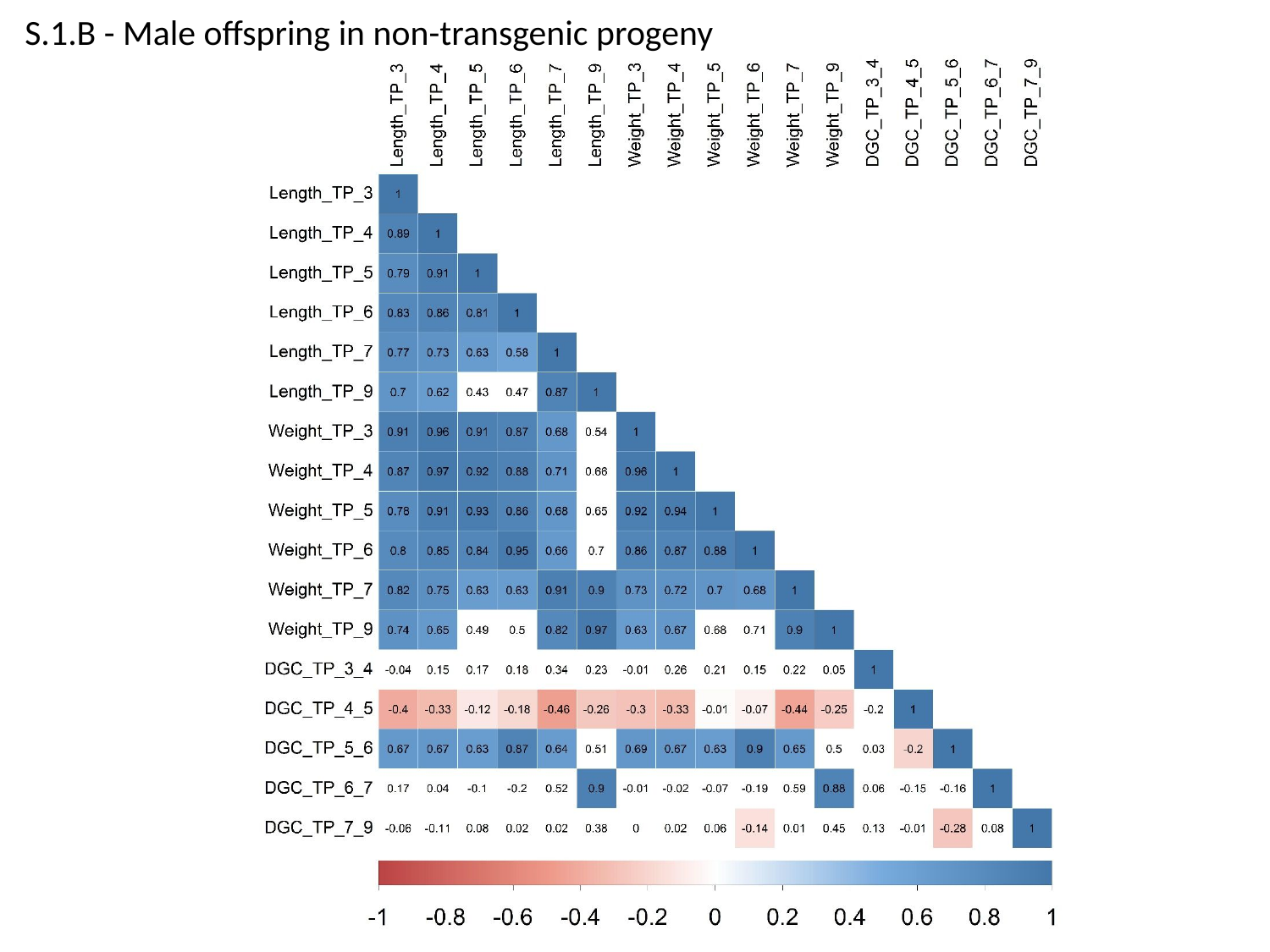

S.1.B - Male offspring in non-transgenic progeny

## Slide 4
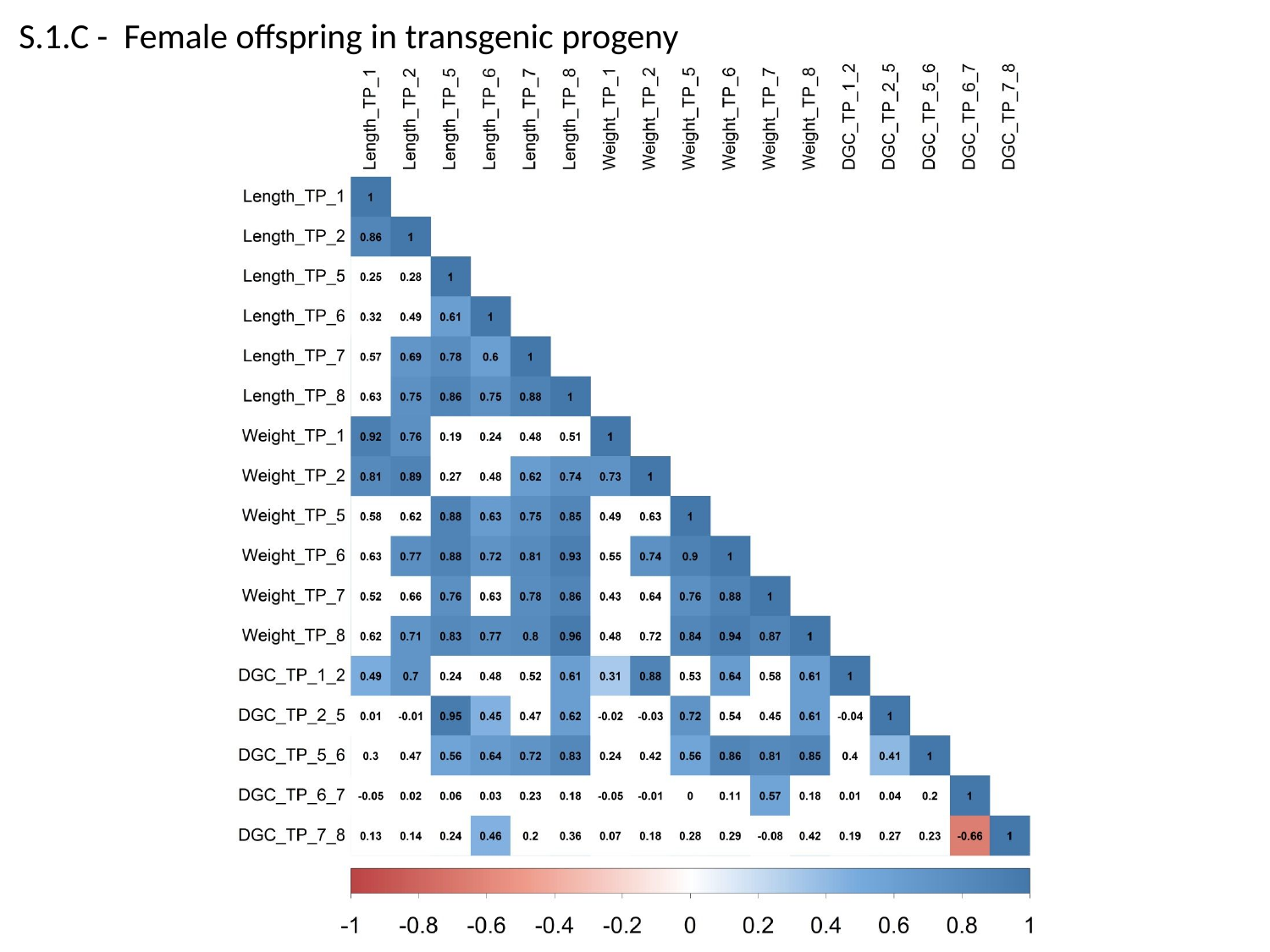

S.1.C - Female offspring in transgenic progeny

## Slide 5
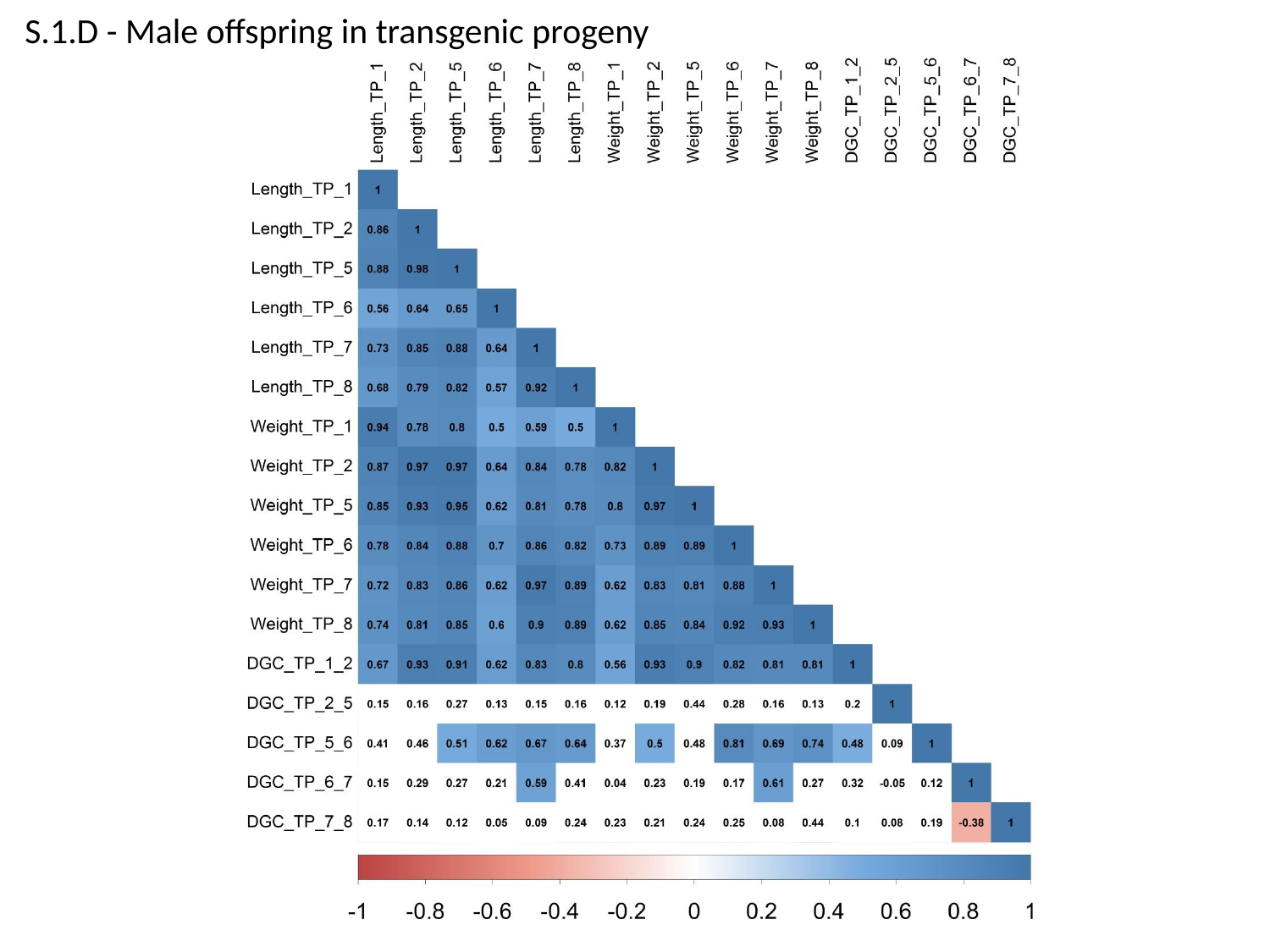

S.1.D - Male offspring in transgenic progeny
