# Supplementary material for: Influence of a growth hormone transgene on the genetic architecture of growth‐related traits: A comparative analysis between transgenic and wild‐type coho salmon
Source: Evol Appl. 2018 Oct 16;11(10):1886–900. doi: 10.1111/eva.12692 (PMC6231474; doi:10.1111/eva.12692)
Supplement: Supplementary file 4 [file EVA-11-1886-s004.pptx]

## Slide 1
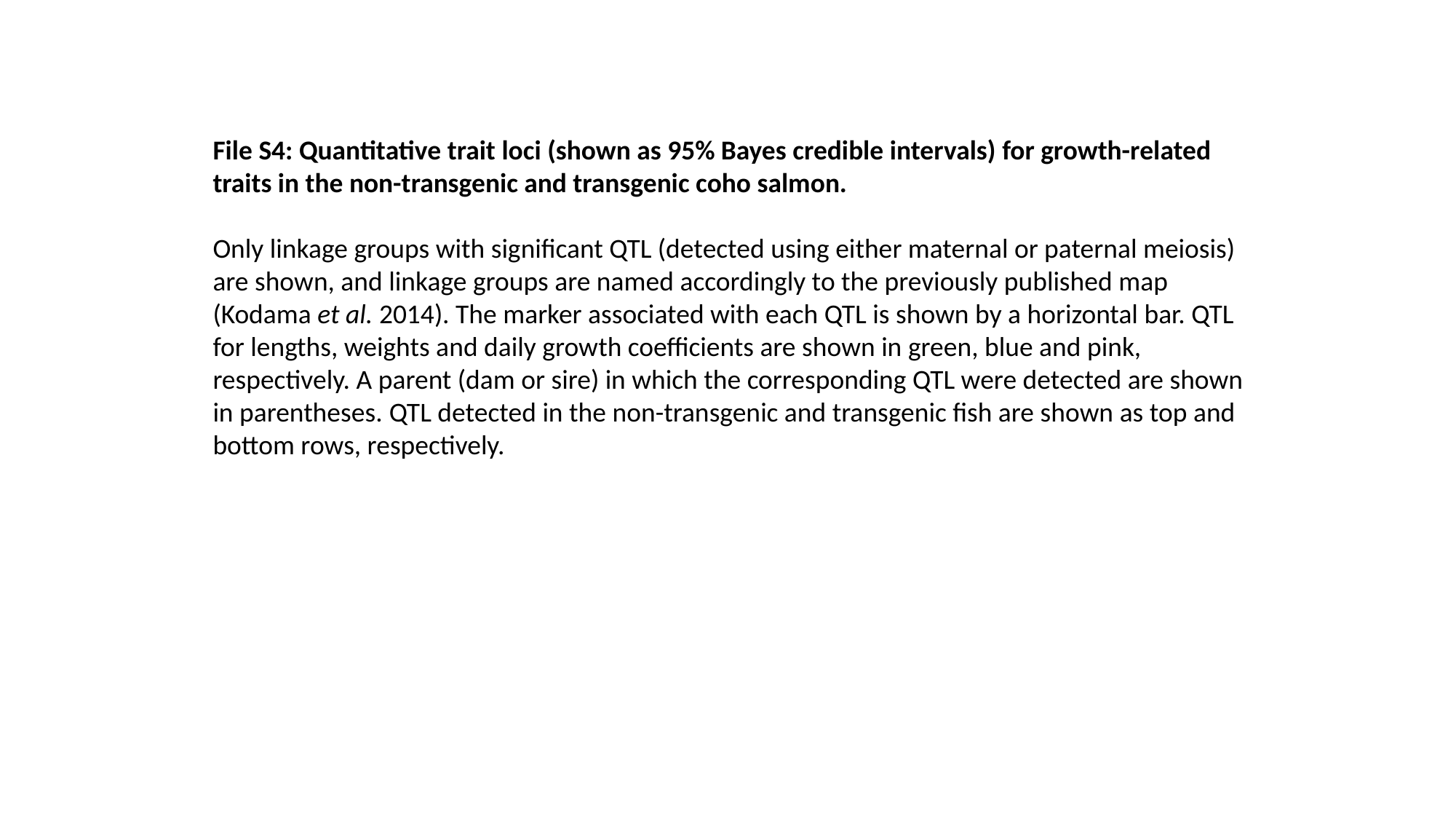

File S4: Quantitative trait loci (shown as 95% Bayes credible intervals) for growth-related traits in the non-transgenic and transgenic coho salmon.
Only linkage groups with significant QTL (detected using either maternal or paternal meiosis) are shown, and linkage groups are named accordingly to the previously published map (Kodama et al. 2014). The marker associated with each QTL is shown by a horizontal bar. QTL for lengths, weights and daily growth coefficients are shown in green, blue and pink, respectively. A parent (dam or sire) in which the corresponding QTL were detected are shown in parentheses. QTL detected in the non-transgenic and transgenic fish are shown as top and bottom rows, respectively.

## Slide 2
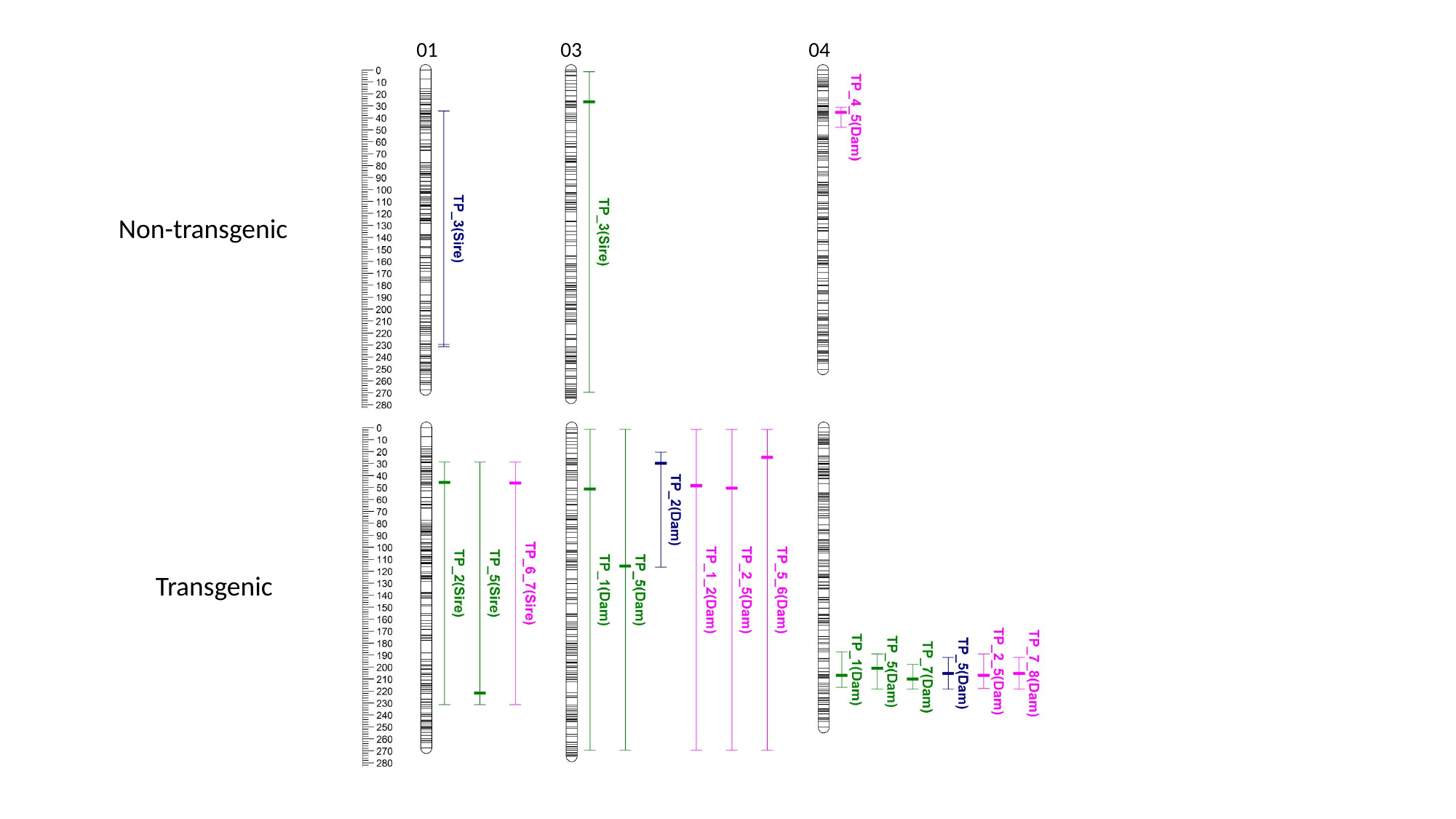

01 03 04
Non-transgenic
Transgenic

## Slide 3
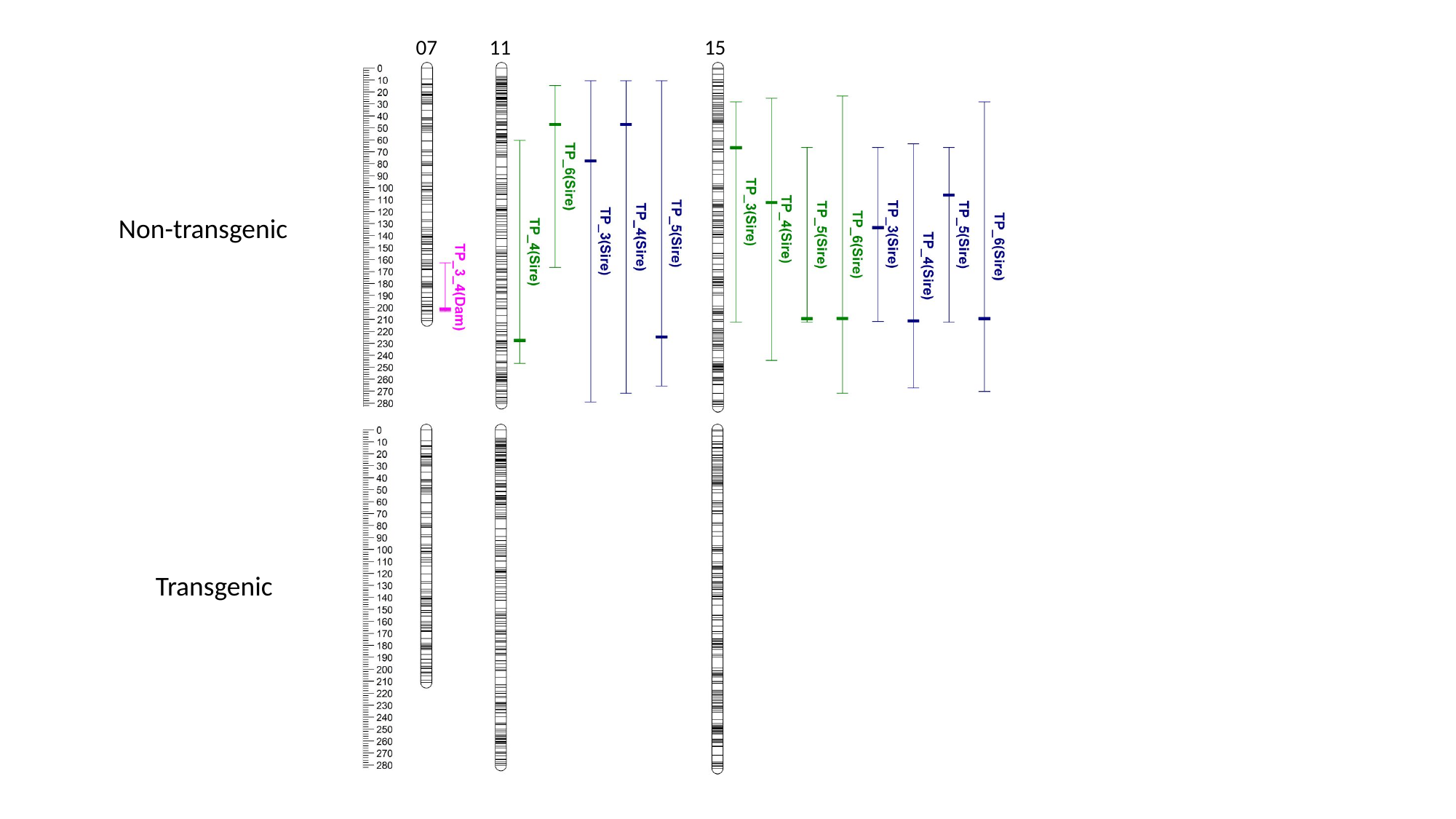

07 11 15
Non-transgenic
Transgenic

## Slide 4
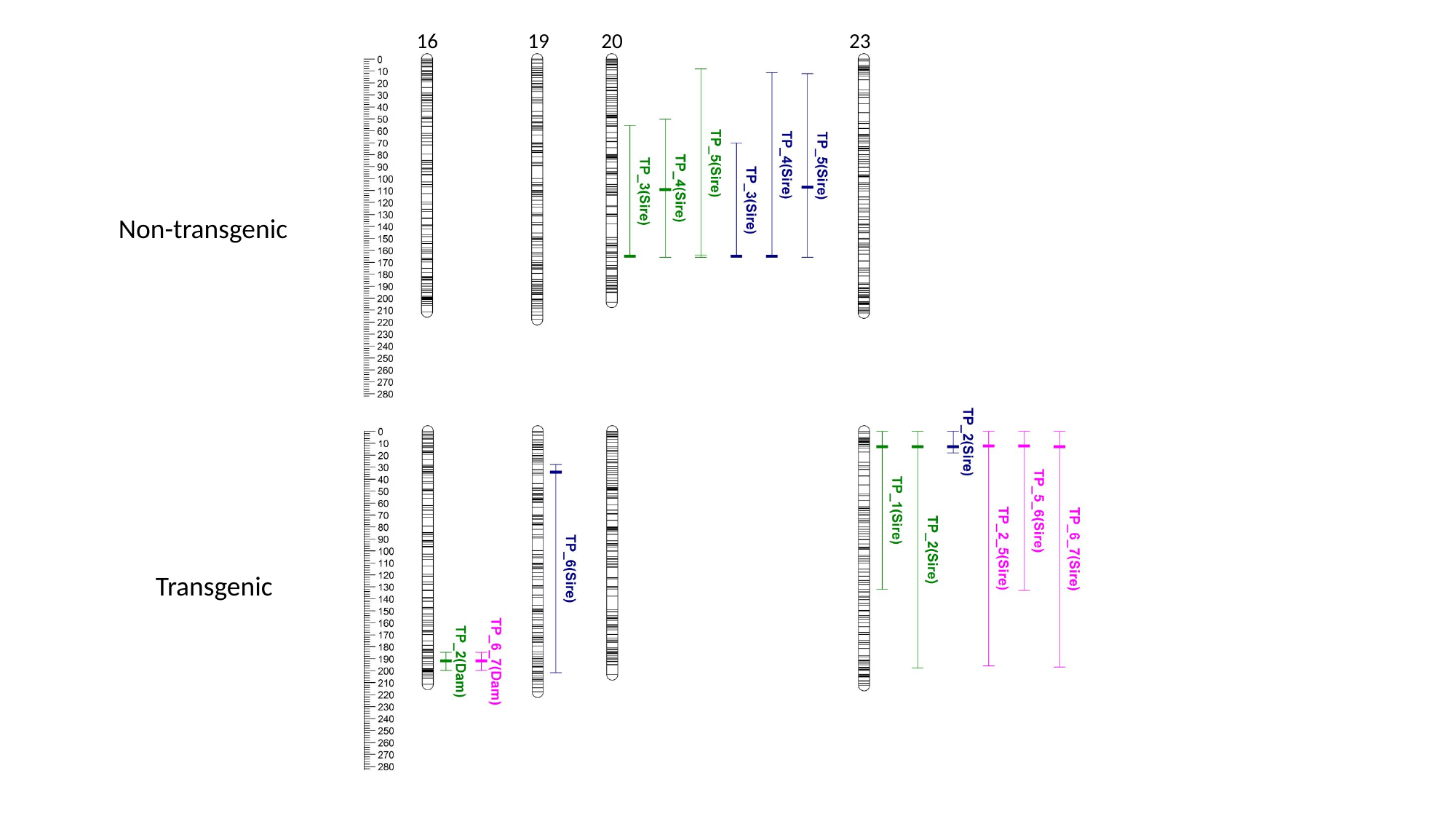

16 19 20 23
Non-transgenic
Transgenic

## Slide 5
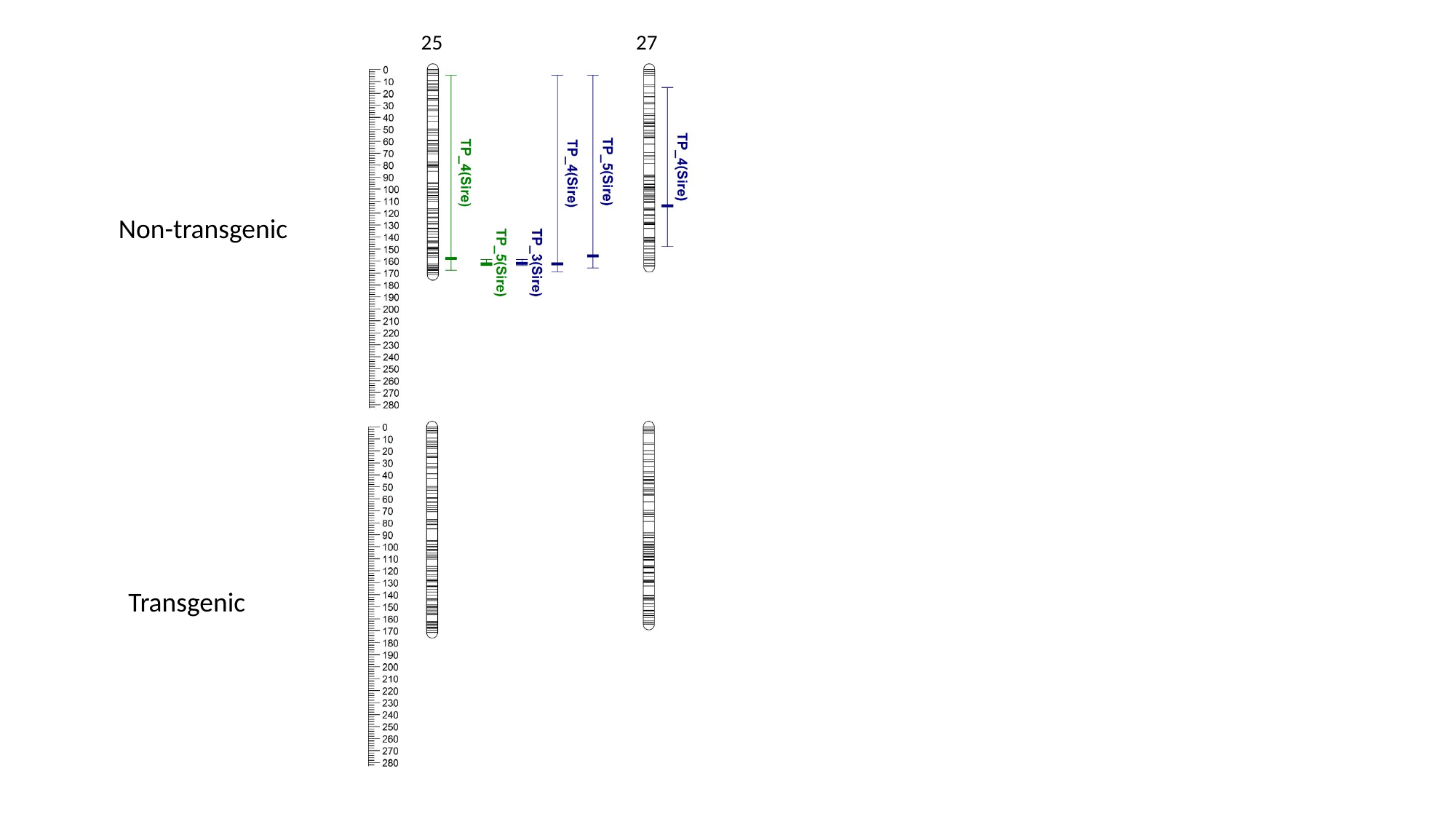

25 27
Non-transgenic
Transgenic

## Slide 6
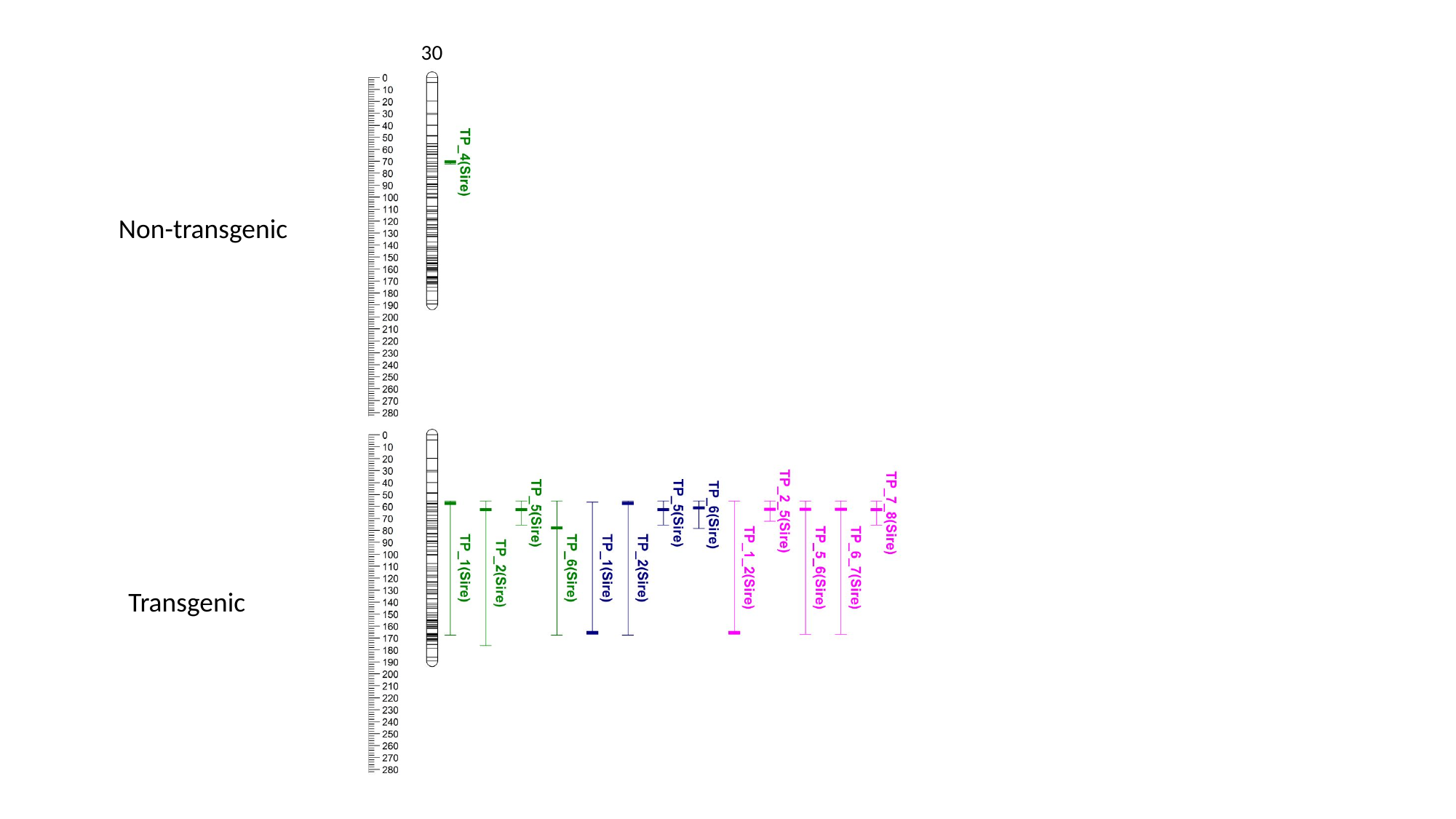

30
Non-transgenic
Transgenic
